# Antecedents and consequences of brand experience in virtual sports brand communities: A value co-creation perspective

**DOI:** 10.3389/fpsyg.2022.1033439

**Published:** 2022-11-23

**Authors:** Jin-Yuan Zhuo, Rong-Hai Su, Hsi-Hsun Yang, Mao-Chou Hsu

**Affiliations:** ^1^Physical Education Department, Renmin University of China, Beijing, China; ^2^College of Physical Education and Sports, Beijing Normal University, Beijing, China; ^3^Department of Digital Media Design, National Yunlin University of Science and Technology, Yunlin, Taiwan; ^4^Department of Recreation and Sports Management, Tajen University, Pingtung, Taiwan

**Keywords:** corporate-initiated value co-creation, customer-initiated value co-creation, brand experience, purchase intention, brand attachment

## Abstract

The influence mechanism for brand experience in virtual sports brand communities is the subject of many studies, but these studies do not feature a holistic consideration of antecedents and consequences, and the moderating role of brand attachment is unclear. Drawing on the value co-creation theory, this study determines the impact of brand experience and its mechanism using the data from 508 virtual sports brand communities. The empirical test results show that value co-creation (i.e., corporate-initiated value co-creation and customer-initiated value co-creation) has a positive effect on the brand experience and that the brand experience has a positive effect on the purchase intention. Brand attachment does not have a moderating role between brand experience and purchase intention so as the degree of brand attachment increases, the brand experience does not increase the purchase intention through a brand attachment. This study determines the antecedents and consequences of brand experience in virtual sports brand communities from a value co-creation perspective, to determine the impact and mechanisms of virtual sports brand communities to guide the marketing practices of virtual sports brand communities.

## Introduction

Information technology allows the people to increase their quality of life via information exchange, communication and interaction, and completing transactions (Zhang et al., 2020; Yang B. et al., 2021). Information technology-based virtual brand communities are platforms that allow consumers to engage with the organizational value creation process and for companies to use consumers to access information (Ranjan and Read, 2021). Virtual sports brand communities are online virtual platforms that allow sports brand enthusiasts to communicate with each other on the theme of a particular sports brand (Ranjan and Read, 2021). The development of information technology has increased the number of virtual brand communities, which provide venues for value co-creation, brand experience, and online shopping and for business-consumer and consumer–consumer interactions (Alimamy et al., 2018). In recent years, traditional value creation theories have been challenged and the concept of value co-creation is increasingly fostered by virtual brand communities (Priharsari et al., 2020). According to the value co-creation theory, the identity of consumers has changed from being value consumers to value creators. Participation in virtual brand community value creation allows consumers to communicate better with companies and to remain updated with current information about companies, products, and services. Furthermore, sharing the experience of using products or services with other consumers improves the brand experience. It also increases customers' willingness to purchase and ultimately enhances the brand value of the corporate entity (Laamanen and Skalen, 2015; Schwetschke and Durugbo, 2018; Rubio et al., 2020a).

With the advent of the experience economy, the focus of companies has shifted from goods or services to customer experience. Consumers' access to a good brand experience increases consumers' brand loyalty and enhances brand equity (Alimamy et al., 2018). Companies seek to enhance the consumers' purchase intention, so that consumers are willing to buy the products or services of these companies. The study of whether value co-creation and brand experience in virtual brand communities can be used to enhance consumers' purchase intention is common (Subbiah and Ibrahim, 2011; Bharti et al., 2015; Yang et al., 2016). Studies on the outcome variables for brand experience focus on the brand loyalty and brand equity, but there is a lack of research on the influence of brand experience on consumer purchase intention and the research mainly involves offline environments. Few studies consider brand experience in virtual brand community environments. There is almost no empirical research on the influence of brand experience on consumer purchase intention. It remains to be verified whether the brand experience that is generated by customers' participation in virtual brand communities and the co-creation of value with companies has a significant impact on purchase intention. This study builds on the results of a previous work to determine the relationship between value co-creation, brand experience, and purchase intention in virtual brand communities.

Attachment fosters a sense of community in consumers and between consumers and companies, by making it easier for them to communicate with each other (Yang M. et al., 2021). Brand relationship study identifies consumers' attachment to brands from the perspective of an emotional connection. Dwivedi et al. (2019) studied the relationship between the influence of consumer emotional attachment on social media consumer brand equity. In a virtual brand community context, the congruence between a corporate entity's message and a customer's self-concept promotes brand attachment (Joshi and Garg, 2021). Consumers with a strong attachment to virtual brand communities engage in more activities, which may involve more posting, reading other people's posts, and other socially relevant behaviors. Brand attachment is an important variable in brand relationship research and its role in influencing brand loyalty and customer purchase intention is of interest in terms of marketing. The stronger the brand attachment, the greater is the consumer's preference for the brand and the greater is the impact on brand equity, which affects the extent to which other variables contribute to brand health (Ansary and Hashim, 2018). Therefore, this study determines whether the relationship between brand experience and purchase intention is affected if brand attachment exists in a virtual brand community.

This study has constructed a theoretical model of the relationship between virtual brand community value co-creation, brand experience, and consumer purchase intention, and determines the moderating role of brand attachment, using a Sports community as the research object to verify the antecedents and consequences of brand experience. The remainder of the paper is organized as follows. Section Literature review and hypotheses presents a literature review, Section 3 details the research methods, and Section 4 details the results of the data analysis, which is followed by a discussion. The final section draws conclusions.

## Literature review and hypotheses

### Value co-creation theory in virtual brand communities

In virtual brand communities, the process of full and effective social interaction between customers and companies, customers and other customer members on product design, development, production, or consumption involves the participation by customers in value co-creation (Zeithaml et al., 2001). The most important element of value co-creation is the customer and the customer's pursuit of maximizing his or her co-creation value dominates all behaviors for the value network (Basole and Rouse, 2008). All activities of the value network are aimed at making value available to the customer (Zeithaml et al., 2001, 2020). Customers are more concerned about the value that is created for themselves than the enterprise value and the co-created customer value is the ultimate reward for customers who engage in value co-creation. For a corporate to enhance its corporate value, it must provide a value proposition to customers or co-create the value that the customers want. Therefore, the goal of the corporate is no longer to create customer value, but to encourage the customers to create the value they need from the services that are provided by the corporate entity, which increases the corporate value. In a virtual sports brand community, customers' participation in value co-creation is complex, between customers and companies and between customers (Leroi-Werelds, 2019). It is necessary to classify the value co-creation and to determine the mechanisms for customer participation in different types of value co-creation (Stampacchia et al., 2020).

Zwass (2010) classified value co-creation in virtual communities into initiated value co-creation and spontaneous value co-creation. This study uses this classification and classifies customer participation in value co-creation into corporate-initiated value co-creation and customer-sponsored value co-creation. Corporate-initiated value co-creation refers to the interaction between customers in new product development activities that are initiated by the corporate entity or the community, such as participation in new product creation, design, evaluation, or promotional activities. Customer-initiated value co-creation refers to customer-initiated interactions with other customers about their experiences with the product.

### Value co-creation and brand experience

In terms of research on brand community experience, in virtual brand communities, the brand experience is the overall experience that an individual receives from his or her interactions with an online brand community, including subjective and internal cognitive, emotional, and social responses in virtual brand communities (Hsu et al., 2010; Nambisan and Watt, 2011; Rose et al., 2012; Lemon and Verhoef, 2016; Wang et al., 2019; Yang B. et al., 2021). Previous studies have considered the dimensions of virtual brand communities as including sensory, emotional, behavioral, and intellectual dimensions, information experience, entertainment experience, homogeneous experience, and relationship experience (Das et al., 2018; Wang et al., 2019; Yang B. et al., 2021). Drawing on previous research in the application context of virtual sports brand communities, this study divides the brand experience into three dimensions: information experience, entertainment experience, and social experience. These dimensions reflect the feelings that customers experience by participating in virtual sports brand community activities.

Through a series of promotional channels, sports companies encourage consumers to participate in virtual brand communities to interact with companies or other consumers. For the initial conceptual and development stages or in the evaluation, trial, and promotional stage, users as potential consumers continue to receive a lot of information and knowledge about the brand (Nysveen and Pedersen, 2014). This information experience is the most direct experience for users who participate in virtual brand communities for value co-creation. Entertainment content is the most basic and important element of virtual brand communities, because it allows companies to display brand- or product-related information in virtual brand communities in diverse forms (including scenes, content, activities, music, and images of the communities), so community users have an entertainment experience (Choi et al., 2016; Ramaswamy and Ozcan, 2016; Biraghi and Gambetti, 2017).

Virtual brand communities use the Internet as a medium to allow users to gather in a community to exchange information about products and this communication with others eliminates loneliness. Companies use virtual brand communities to gather a group of people with similar experiences to communicate with each other and to create intrinsic social connections (Merz et al., 2018). This experience is the interactive experience. Previous studies have shown that consumer–company interactions directly and positively influence customer experience (Nobre and Ferreira, 2017). Therefore, this study proposes the following hypotheses.

H1a: Corporate-initiated value co-creation has a positive effect on entertainment experience.
H1b: Corporate-initiated value co-creation has a positive effect on information experience.
H1c: Corporate-initiated value co-creation has a positive effect on sociability experience.

Virtual sports brand communities serve as a platform for value co-creation between customers and companies or other customers. Community users interact with one another to obtain information about the products and brands and to have an informative experience in terms of brand knowledge (Rubio et al., 2020b). This information is presented in the form of pictures, images, and Flash and the users communicate with each other about the features or uniqueness of the product, generating visual and auditory reactions and positive feelings, which enhance the entertainment experience (Zhao et al., 2019). Interactive experiences occur when interpersonal interactions occur (Fuller and Bilgram, 2017), when the consumers spontaneously participate in the value co-creation process in virtual brand communities, and when the consumers interact with consumers within the community to create certain social relationships with each other (Hsieh, 2015). Consumers in virtual brand communities sacrifice their independence as individuals to foster a connection to other consumers, so the interactive experience is enhanced (Rubio et al., 2020b). Based on the above discussion, this study proposes three research hypotheses as follows.

H2a: Customer-initiated value co-creation has a positive effect on entertainment experience.
H2b: Customer-initiated value co-creation has a positive effect on information experience.
H2c: Customer-initiated value co-creation has a positive effect on sociability experience.

### Brand experience and purchase intention

During value co-creation activities, the customers discuss and communicate with companies and other customers about products and the platform of a virtual network brings consumers a new shopping experience, which alters their feelings about the corporate entity and the brand and creates a different brand experience (Ul Islam et al., 2017). Prior research on brand experience is common in the field of brand management (Chang and Hsu, 2022) and interactive communication in virtual sports brand communities changes the brand experience of consumers.

Stimulating consumers' emotions through virtual brand communities allows them to understand and recognize the brand better and to create a personal brand image (Naylor et al., 2012). As consumers experience the brand more meaningfully, the degree of brand association increases (Keng et al., 2016). This development generates or increases consumers' purchase intention (Wu and Hsu, 2018). Brand experience also influences consumers' purchase intention through four intermediate variables: self-perception consistency, brand attitude, subjective norms, and perceived behavioral control (Chakraborty, 2019; Kumar, 2022). Therefore, research hypotheses are proposed as follows.

H3a: Entertainment experience has a positive effect on purchase intention.
H3b: Information experience has a positive effect on purchase intention.
H3c: Sociability experience has a positive effect on purchase intention.

### The moderating role of brand experience

Attachment is a relational construct that influences or shapes the level of consumer brand commitment (or brand loyalty) (Bowlby, 1969; Yang M. et al., 2021; Yang et al., 2022). In terms of the various factors that influence consumers' emotional attachment to social media brands in brand relationships, consumers focus on the entertainment experience, the information experience, and the sociability experience. Consumers also have more opportunities to experience the virtual sports brand community during the interaction process, which leads to a sense of belonging and emotional attachment to the sports brand. This increases purchase intention for the sports brand.

Studies show that users' views of brand pages and purchase intentions are influenced by product selection, personalized ads, and related online activities such as brand messages and by brand attachment (Enginkaya and Yilmaz, 2014). The greater the level of emotional attachment. The more willing are consumers to approach the brand and maintain a lasting relationship with the brand, the higher is the perception of the object of attachment and the willingness to pay a premium. Therefore, consumers are less likely to seek substitutes and more likely to exhibit brand loyalty (Bowlby, 1982), which sustains purchase intention. The greater the level of brand attachment, the better is the consumer brand experience and competing brands become less attractive to the consumers, which increases consumer purchase intention. This study proposes the following hypotheses:

H4a: Brand attachment plays a moderating role between entertainment experience and purchase intention.
H4b: Brand attachment plays a moderating role between information experience and purchase intention.
H4c: Brand attachment plays a moderating role between sociability experience and purchase intention.

**Figure 1:**
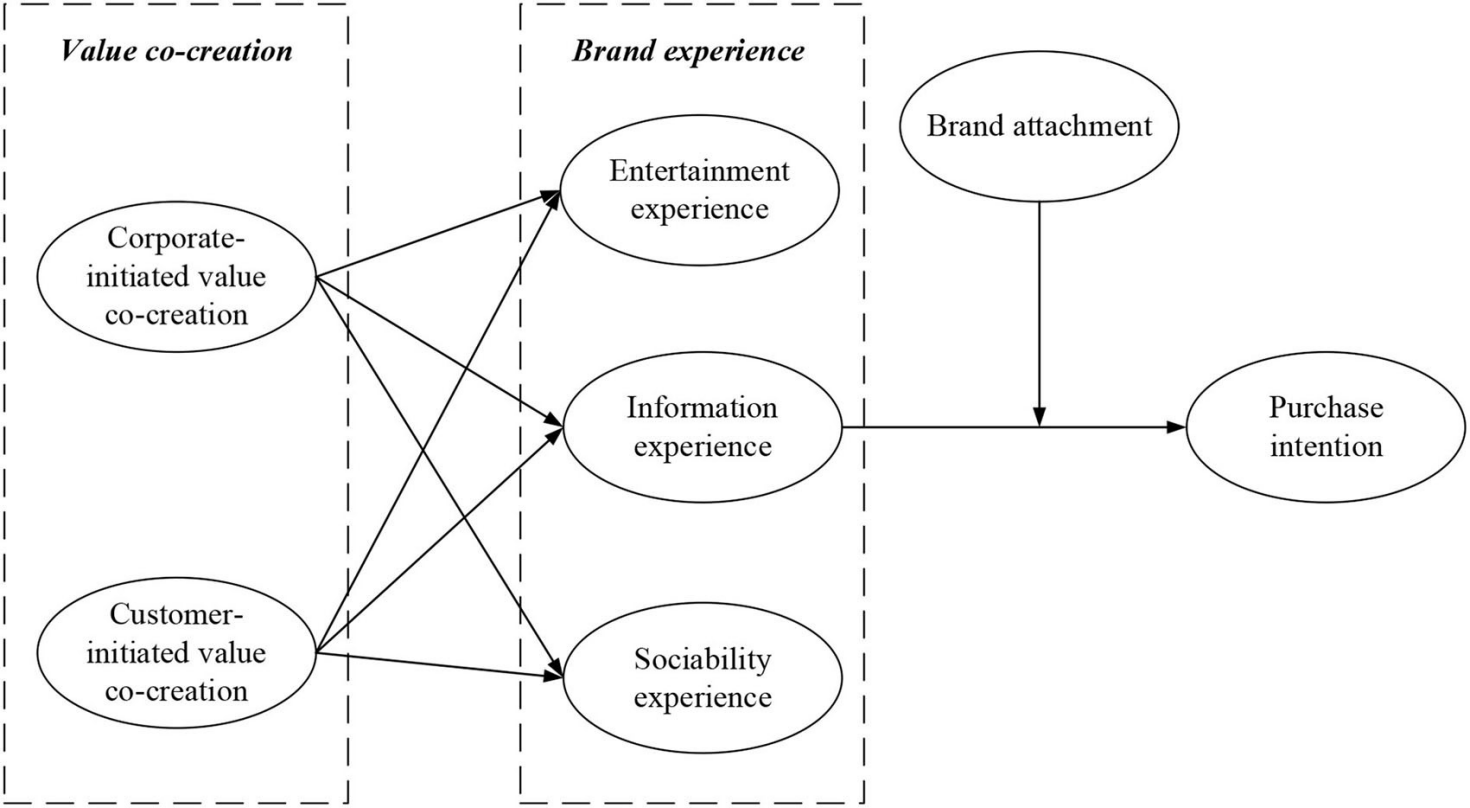
Theoretical model.

The research model for this study is shown in Figure 1.

## Research methods

### Measures

This study measures corporate-initiated value co-creation, customer-initiated value co-creation, entertainment experience, information experience, sociability experience, brand attachment, and purchase intention using established scales for structural equation modeling (SEM). The measurement questions were rewritten to fit the research context, so all variables were measured to fit the conditions for this study. A Likert scale (1 = strongly disagree, 7 = strongly agree) is used to measure the question items for all variables. The specific details of the variables that are measured in this study are shown in Table 1.

**Table 1:** Variables and measurement items.

**Variables**	**Items**	**Sources**
Corporate-initiated value co-creation	COVC1. I regularly participate in community-sponsored product idea calls or evaluations. COVC2. I regularly participate in community-sponsored product design calls or evaluations. COVC3. I regularly participate in community-sponsored product reviews. COVC4. I often participate in community-sponsored product promotion activities.	Zwass, 2010
Customer-initiated value co-creation	CUVC1. I often share my experience of using the brand with members in the community. CUVC2. I often start brand or product-related topics in the community. CUVC3. I often respond to other members' topics in the community. CUVC4. I often help other members in the community to solve their problems	Zwass, 2010
Entertainment experience	EE1. I think the community has interesting content. EE2. I think the community allows me to relax and feel happy. EE3. I think the community relieves me of stress.	Nambisan and Watt, 2011; Wang et al., 2019
Information experience	IE1. I can get some useful information or resources in the community. IE2. I can provide the community with information that others need. IE3. I can find some solutions to problems in the community.	Nambisan and Watt, 2011; Wang et al., 2019
Sociability experience	SE1. In the community, I can get positive feedback from other users. SE2. In the community, I can communicate better with other users. SE3. In the community, I can impress other users.	Nambisan and Watt, 2011; Wang et al., 2019
Brand attachment	BA1. I have a relationship of dependence on the brand. BA2. I feel close to the brand when I see it. BA3. I am always enthusiastic and excited about the clothing brand. BA4. Seeing the brand reminds me of things related to it	Thomson et al., 2005
Purchase intention	PI1. I would recommend this sports livestreaming platform to my friend. PI2. I would buy the product or service of the sports livestreaming platform. PI3.There is a probability that I would consider buying the product or service of the sports livestreaming platform.	Pavlou, 2003; Lin et al., 2011; Wang et al., 2019

### Samples and data collection

For this study, the subjects of the questionnaire were virtual sports brand community users and an online questionnaire was used. Screening produced more representative subjects, which also increases the veracity of the conclusions of this study. The study collected questionnaires by posting questionnaire information on a professional questionnaire website (https://www.wjx.cn/) and informing the consumers who had used the virtual sports brand community before completing the questionnaire. To improve the quality of the questionnaire, 5 RMB (renminbi) was given to the subjects. Members of the virtual sports brand community also saw a link to the questionnaire in the community and inviting community members to complete it. The questionnaire stated that a reward of 5 RMB was applicable. The questionnaire was available from February 22 to May 30, 2022 and a total of 528 questionnaires were collected. The number of actual valid questionnaires was 508 and the questionnaire efficiency rate was 96.21%. There were as many male as female respondents (50%, of the total 254), with 327 married users (64.4%), 177 unmarried users (34.8%), and 4 divorced users (0.8%). In terms of continuous use time, the least percentage of the pack was less than 1 year (73 respondents), while the most percentage of the pack was over 3 years (222), and the middle of the pack was 1–2 years (213). In terms of age characteristics, 30–39 years (220 participants) ranked first with 43.3%, followed by 20–29 years ranked second (209 participants) with 41.1%, 40–49 years ranked third (47 participants, 9.3%), 50 or above years, ranked fourth (22 participants, 4.3%), and 19 or less years ranked the least. There were only 10 respondents. Under the Education category, 77.6% of the respondents were undergraduate, 14.0% were college and below, and 8.5% held master's degree and above. In terms of consumption, 6,000 or more RMB (55.1%) was the most popular, followed by 4,000–5,999 RMB (22.4%) and 2,000–3,999 RMB (15.4%), and below 2,000 RMB (7.1%) was the least popular.

## Data analysis

### Measurement model

Partial least squares (PLS) was applied to determine the antecedents and consequences of brand experience in virtual sports brand communities of this study. This study follows previous research which had suggested to evaluate the measurement model analysis to determine whether a measurement model has acceptable convergent validity (Hair et al., 2017). In this study, the range of factor loadings for all dimensions is from 0.764 to 0.932, Cronbach's alpha is from 0.835 to 0.882, composite reliability (CR) is from 0.887 to 0.928, rho_A is from 0.843 to 0.914, and average variance extracted (AVE) is from 0.663 to 0.811. The results of factor loading, Cronbach's Alpha, CR, rho_A, and AVE meet the suggestions of Hair et al. (1998), Nunnally and Bernstein (1994), and Fornell and Lacker (1981). The results in Table 2 show that the results of the confirmatory factor analysis (CFA) indicate good convergence validity for all variables.

**Table 2:** Confirmatory factor analysis (CFA).

**Variables**	**Item**	**Factor loadings (*t*-values)**	**CR**	**Cronbach's Alpha**	**Rho_A**	**AVE**
Corporate-initiated value co-creation	COVC1	0.764 (24.578)	0.894	0.845	0.874	0.680
	COVC2	0.780 (25.018)				
	COVC3	0.878 (62.369)				
	COVC4	0.870 (57.831)				
Customer-initiated value co-creation	CUVC1	0.766 (26.743)	0.892	0.838	0.843	0.673
	CUVC2	0.840 (43.340)				
	CUVC3	0.846 (44.512)				
	CUVC4	0.827 (42.891)				
Entertainment experience	EE1	0.907 (90.165)	0.907	0.846		0.765
	EE2	0.870 (65.085)				
	EE3	0.846 (49.885)				
Information experience	IE1	0.830 (43.042)	0.899	0.835	0.847	0.749
	IE2	0.890 (67.561)				
	IE3	0.875 (81.811)				
Sociability experience	SE1	0.862 (60.181)	0.904	0.842	0.857	0.760
	SE2	0.919 (99.080)				
	SE3	0.831 (39.340)				
Brand attachment	BA1	0.868 (5.169)	0.887	0.844	0.914	0.663
	BA2	0.787 (5.010)				
	BA3	0.865 (6.056)				
	BA4	0.730 (3.613)				
Purchase intention	PI1	0.925 (73.907)	0.928	0.882	0.884	0.811
	PI2	0.932 (86.192)				
	PI3	0.841 (34.062)				

Table 3 lists the means and standard deviations for the variables, the discriminant validity for the measurement model, and the square roots of the AVE that are on the diagonal. Discriminant validity is the extent to which the measure is not a reflection of other variables. This study determines discriminant validity using Fornell and Lacker (1981)'s recommendation. Table 3 shows that the squared root of average variance for each construct is greater than the correlations between the constructs and all other constructs. These results support Fornell and Lacker (1981)'s requirement for discriminant validity.

**Table 3:** Discriminant validity, means, and standard deviations.

**Variables**	**1**	**2**	**3**	**4**	**5**	**6**	**7**	**Mean**	**Standard deviation**
COVC	***0.825***							5.157	1.139
CUVC	0.479	***0.821***						4.902	1.235
EE	0.360	0.407	***0.875***					4.453	1.317
IE	0.399	0.343	0.615	***0.865***				4.295	1.291
SE	0.344	0.309	0.528	0.671	***0.872***			4.028	1.390
BA	0.330	0.309	0.229	0.277	0.197	***0.814***		4.954	1.151
PI	0.449	0.401	0.537	0.514	0.443	0.150	***0.901***	5.747	1.118

COVC, corporate-initiated value co-creation; CUVC, customer-initiated value co-creation; EE, entertainment experience; IE, information experience; SE, sociability experience; PI, purchase intention. The bold values in diagonal values are the square root of AVE.

### Structural model

Table 4 shows the results of a path model testing hypothesized effects. Hypotheses 1a, 1b, and 1c discussed the relationship of corporate-initiated value co-creation and entertainment experience, information experience, with sociability experience. Company-initiated value co-creation was indeed found to be significantly associated with entertainment experience (β = 0.214, *p*-value < 0.001), information experience (β = 0.305, *p*-value < 0.001), whereas sociability experience was also found (β = 0.255, *p*-value < 0.001). H1a and H1b were thus supported, whereas H1c was also supported. The results of the empirical study show that value co-creation in virtual sports brand communities that is initiated by companies has a significant positive impact on consumers' information experience, entertainment experience, and interactive experience. Customer-initiated value co-creation is positively associated with entertainment experience (β = 0.305, *p* < 0.01), information experience (β = 0.197, *p*-value < 0.01), and sociability experience (β = 0.187, *p*-value < 0.01). Therefore, H2a, H2b, and H2c are supported.

**Table 4:** Path coefficient estimates.

**Path**	**β**	**SD**	***T***	**Result**
H1a: COVC->EE	0.214^***^	0.058	3.705	Accepted
H1b: COVC->IE	0.305^***^	0.068	4.509	Accepted
H1c: COVC->SE	0.255^***^	0.064	3.975	Accepted
H2a: CUVC->EE	0.305^***^	0.058	5.237	Accepted
H2b: CUVC->IE	0.197^**^	0.065	3.014	Accepted
H2c: CUVC->SE	0.187^**^	0.064	2.905	Accepted
H3a: EE->PI	0.334^***^	0.05	6.641	Accepted
H3b: IE->PI	0.235^***^	0.049	4.774	Accepted
H3c: SE->PI	0.109^**^	0.042	2.623	Accepted

** *p*-value < 0.01,

*** *p*-value < 0.001.

The results of this study show that customer-sponsored virtual sports brand community value co-creation has a significant positive impact on consumer information experience, entertainment experience, and interactive experience. Entertainment experience (β = 0.334, *p* < 0.001), information experience (β = 0.235, *p*-value < 0.001), and sociability experience (β = 0.109, *p*-value < 0.01) are positively associated with purchase intention. Therefore, H3a, H3b, and H3c are supported. The results also show that an improvement in consumers' entertainment, information, and interaction experience during value co-creation in a virtual sports brand community significantly contributes to increased purchase intention.

The moderating effects are listed in Table 5. For this study, brand attachment is the moderating variable. The results of structural equation modeling (SEM) show that entertainment experience × brand attachment has a moderating effect on the purchase intention of 0.027 (*T* < 1.96, *p* > 0.05), so brand attachment has a positive moderating effect on the relationship between entertainment experience and purchase intention. Specifically, the gradient of entertainment experience against purchase intention increases positively by 0.027 units for each 1-unit increase in the moderating variable brand attachment. That is, brand attachment does not have a positive moderating effect. Therefore, H4a is not supported. Similarly, H4b and H4c are also not supported.

**Table 5:** Analysis of moderating effect.

**Dependent variable**	**Independent variable**	**β**	***T***	***p***	**Result**
Purchase intention	Entertainment experience	0.333^***^	6.013	^***^	Not supported
	Brand attachment	0.015	0.266	ns	
	Entertainment experience × brand attachment	0.027	0.413	ns	
Purchase intention	Information experience	0.249^***^	4.367	^***^	Not supported
	Brand attachment	0.015	0.266	ns	
	Information experience × brand attachment	0.013	0.230	ns	
Purchase intention	Sociability experience	0.100	1.940	ns	Not supported
	Brand attachment	0.015	0.266	ns	
	Sociability experience × brand attachment	0.058	1.150	ns	

*** *p*-value < 0.001; *ns*, nonsignificant.

## Results and discussion

### Discussion

Corporate-initiated value co-creation has a significant positive effect on brand experience. In terms of the consumer, enterprises initiate value co-creation behaviors to create value with consumers and promote their brands, products, or services. Therefore, virtual sports brand community value creation activities that are initiated by companies, such as new product design and idea collection, stimulate customers' curiosity and desire to participate, so they actively give their opinions. The virtual sports brand community value creation activities that are initiated by enterprises are based on their own development needs and involve a series of value co-creation activities. This incorporates the needs and interests of consumers and promotes participation in the process of value co-creation by participating in activities, exchanging experiences, and making suggestions, which involves positive value co-creation for consumers and has a positive impact on their entertainment experience and interactive experience.

Customer-initiated value co-creation has a significant positive impact on brand experience. In terms of spontaneous participation in the virtual sports brand community for value co-creation, active interaction between customers increases access to information and the accuracy of the information obtained, which increases the information experience of consumers, because these factors rely on independent activities in the virtual sports community, including experience sharing and helping other customers to solve their problems and resolve doubts. Spontaneous participation in virtual sports brand communities allows consumers to express their own opinions on content that interests them, which significantly enhances their entertainment experience. Interaction between customers also promotes the formation of social networks, improves the emotional connection between customers and user stickiness, and enhances the consumer interaction experience.

The effect of brand experience (entertainment experience, information experience, and interactive experience) on purchase intention is determined. In the virtual brand community, community users are potential consumers, so they exchange product usage experiences and solve product-related problems with other users or companies in the community. Online communication allows users to interact and communicate in a timely and personalized manner. This feature creates a sense of belonging to the virtual brand community, which increases the customer's entertainment and interactive experience. These brand experiences allow consumers to recognize, satisfy, and trust the product, corporate entity, and brand, so purchase intention is increased. In the virtual brand community, customers interact by participating in product production, design, and evaluation or by sharing consumption experience with corporate customers, so customers have a different consumption experience and brand experience to that which is available in an offline shopping environment. This encourages value co-creation and benefits both consumers and companies.

The moderating effect of brand attachment is not examined. Brand attachment is a moderating variable in this study because the level of brand attachment affects the performance of customers in terms of consumption behavior. The results for the moderating effect show that brand attachment does not play a significant moderating role in the effect of brand attachment on current purchase intention. For higher levels of brand attachment, the influence of brand experience on customers' purchase intention is not increased. The strong emotional bond of brand attachment means that consumers with a higher level of brand attachment are not given more attention, so there are no economic benefits or intangible assets for the brand. This means that virtual sports brand communities cannot use their immediacy to respond quickly to consumer needs and give feedback to enhance the customer's experience of the brand through emotional or demand communication and increase purchase intention.

### Theoretical contributions

This study is similar to the study by Vargo and Lusch (2008), who noted that marketing logic is evolving from a commodity-led logic to a service-led logic, and the study by Merz et al. (2018), which notes that brand value is co-created by corporations and stakeholders. This study focuses on how consumer-firm value co-creation affects consumers' purchase intention in a virtual sports brand community environment and determines whether this process is moderated by brand attachment. This study assumes that the role of customers is shifting from value consumers to value co-creators and uses virtual sports brand communities as a platform for value co-creation to empirically study the process and results of value co-creation. The results of this study are relevant to the value co-creation theory in terms of service-oriented logic.

This study determines the moderating effect of brand attachment on the relationship between brand experience and purchase intention. Studies show that the greater the level of brand attachment, the greater is the influence of changes in entertainment experience and interactive experience on purchase intention. At the same time, the lower the level of brand attachment, the lesser is the influence of changes in entertainment experience and interactive experience on purchase intention (Dwivedi et al., 2019).

The results of this study show that consumer brand attachment regulates the relationship between entertainment experience, interactive experience, and purchase intention. Therefore, it is not necessary for companies to consider the role of brand attachment in facilitating purchase intentions after the interaction between the virtual sports brand community's platform and consumers generates co-created value and enhances consumers' brand experience. It is directly through the brand experience that consumers' purchase intentions are stimulated to reach a consumer decision and achieve maximum purchase intention.

### Practical implications

Companies must abandon the logic of brand marketing based on the idea of value co-creation. Consumers are present in all processes involving value production and the brand value is ultimately determined by consumers, so only by consumer recognition can the brand can be more valuable (Zwass, 2010). Therefore, brand marketing must consider the importance of the role of consumers in creating value in order to create a strong brand. Compared with customer-initiated virtual sports brand community value co-creation, it is easier for companies to control and operate their own virtual sports brand community value co-creation (Zeithaml et al., 2020). When companies initiate virtual sports brand community value co-creation, they must consider the management and supervision of their communities and actively address user needs in order to design more attractive community applications, interactive community activities, and brand-related community topics (Alimamy et al., 2018). Users' brand experience is improved by providing real and effective brand information to users, so that every community user can easily get relevant brand information and have fun in the process of value co-creation (Biraghi and Gambetti, 2017). In the process of interacting with other users in the virtual sports brand community, an interpersonal network enhances the overall information experience, entertainment experience, and interactive experience.

Companies must emphasize the role of virtual sports brand communities as a platform for value co-creation. A virtual sports brand community is a product of the continuous advancement of online technology and is constantly valued because it enhances marketing as a value co-creation platform for business development. In the era of the experience economy (Enginkaya and Yilmaz, 2014), companies must use this platform to allow consumers to improve their consumption experience and increase consumers' purchase intention, which benefits the core competitiveness of enterprises. The results of this study show that the brand experience effects of corporate-initiated and customer-initiated value co-creation behaviors are not the same. Only customers' spontaneous participation in the value co-creation process improves their brand experience, mainly due to the dissemination of corporate disinformation and the bias of consumers (Zeithaml et al., 2020). Therefore, companies need not design and launch value co-creation activities. By adopting the perspective of consumers, they can provide the real and effective information based on their needs and design value co-creation activities that attract new members and help them communicate better with each other. When consumers communicate better with each other and understand the product knowledge better, their interest in the brand is fostered.

Consumer purchase intention is increased by participation in virtual sports brand communities. The results of this study show that value co-creation in customer-initiated virtual brand communities enhances all dimensions of consumers' brand experience (Wang et al., 2019), including information experience, entertainment experience, and interactive experience and consumers' purchase intention. Managers of a virtual sports brand community must ensure that each contact point for interaction with customers has a positive environment and that the person who manages each section of the virtual brand community is professionally trained, so that they can provide timely, effective, and high-quality services with customers as the center (Leroi-Werelds, 2019). The advent of the era of the experience economy and value co-creation has highlighted the role of customer experience and value co-creation (Rubio et al., 2020a). As a platform for brand value co-creation, virtual sports brand communities allow enterprises to remain competitive and promote long-term stable development. Companies must cultivate virtual brand communities and encourage and support consumers' spontaneous value co-creation activities by ensuring that the operation and management of virtual sports brand communities uses a strategy of corporate brand management.

## Limitations and future directions

The sample from which data were collected for this study includes individuals between 20 and 39 years, representing 84.4% of the overall sample. Future research might include the under-20 and over-40 age groups, as these two groups are also part of the main market for Virtual Sports Brand Communities. Future research might also determine the differences in the antecedents and consequences of the brand experience for these two groups. Second, the data for this study's questionnaire are for the same point in time, so there may be a lack of common method variance. Future research should use dynamic data for validation and might use experimental methods or qualitative research that involves dynamic attitudinal data.

## Data availability statement

The raw data supporting the conclusions of this article will be made available by the authors, without undue reservation.

## Author contributions

Conceptualization: J-YZ, R-HS, and H-HY. Formal analysis: H-HY and M-CH. Investigation: J-YZ and R-HS. Writing original draft: J-YZ, R-HS, H-HY, and M-CH. Writing–review and editing: J-YZ, R-HS, H-HY, and M-CH. All authors contributed to the article and approved the submitted version.

## Conflict of interest

The authors declare that the research was conducted in the absence of any commercial or financial relationships that could be construed as a potential conflict of interest.

## Publisher's note

All claims expressed in this article are solely those of the authors and do not necessarily represent those of their affiliated organizations, or those of the publisher, the editors and the reviewers. Any product that may be evaluated in this article, or claim that may be made by its manufacturer, is not guaranteed or endorsed by the publisher.
